# Prevalence of perceived discrimination, determinants and associations with self-rated general and sexual health, healthcare utilization and self-perceived integration: a cross-sectional survey of migrants in Sweden

**DOI:** 10.1186/s12889-024-18160-2

**Published:** 2024-03-05

**Authors:** Faustine Kyungu Nkulu Kalengayi, Mazen Baroudi, Anna-Karin Hurtig

**Affiliations:** Department of Epidemiology and Global Health, Umeå University, Umeå, SE-901 87 Sweden

**Keywords:** Discrimination, Self-rated health, Sexual health, Healthcare access, Social integration, Migrants/immigrants, Ethnicity, Religion, Integration paradox, Sweden

## Abstract

**Background:**

Sweden has welcomed migrants, but attitudes have shifted, becoming hostile due to populism and the growing number of migrants. This has left migrants feeling unwelcome and marginalized. Few studies have examined the extent to which migrants perceive discrimination, who, why, where and its relationships with different outcomes. This study has two aims: to assess the prevalence, reasons, and determinants of perceived discrimination among migrants (1) and its associations with self-rated health, sexual health, healthcare use, and integration (2).

**Methods:**

We analysed data from a 2018 survey on migrants’ sexual and reproductive health and rights. The survey included 1740 migrants aged 16 or older. We used descriptive and log-binomial regression analyses to estimate prevalence, crude and adjusted prevalence ratios (APR) with 95% confidence interval (CI).

**Results:**

About 36% of participants perceived discrimination in Sweden, with ethnic origin (62%) and religion (35%) as main reasons. Perceived discrimination occurred in public spaces (47%), schools (33%), internet (20%), work (19%), public services (18%), residential areas (16%), and healthcare settings (10%). Migrant men (APR: 1.26, CI:1.07–1.49), born in Middle East and North Africa (APR: 1.57, CI:1.26–1.95) and South Asia (APR: 1.61, CI:1.27–2.04) regions, with more than 12 years of education (APR: 1.33, CI:1.10–1.60), a non-heterosexual orientation (APR: 1.21, CI: 1.02–1.43), a non-Christian religion (APR: 1.41, CI: 1.10–1.80), economic stress (APR:1.67, CI: 1.44–1.93) or Swedish language skills (APR: 1.24, CI:1.07–1.43) perceived discrimination more than their counterparts. In contrast, the oldest participants (46 years or more) perceived less discrimination (APR:0.55, CI: 0.37–0.80) than the youngest ones (16–25 years). Moreover, perceived discrimination was associated with poor self-rated general (APR:1.72, CI: 1.45–2.04) and sexual health (APR:1.40, CI:1.2–1.64), integration (APR:1.25, CI:1.14–1.37), and healthcare access (APR: 1.48, 1.16–1.89).

**Conclusions:**

This study shows that migrants in Sweden face widespread perceived discrimination based on ethnicity and religion. This can affect their health, healthcare use, and social integration. The study calls for policies and interventions that tackle systemic perceived discrimination, foster inclusion, and guarantee equal opportunities in accessing healthcare and resources for migrants. It also urges support for vulnerable groups who perceive more discrimination, such as migrants from certain regions or under economic stress.

## Background

The number of migrants living in Sweden has significantly increased in the last few decades [1]. Statistics Sweden defines ‘migrant’ as everyone who moves to Sweden and becomes registered by the Swedish tax authorities (*Skatteverket*). To be registered, non-citizens must have the intention and legal right to stay in Sweden for at least twelve months. However, the rules differ for different migrant groups. For instance, while Nordic citizens can freely migrate to Sweden, Citizens from other countries of the European Union (EU)/European Economic Area (EEA) are required to meet the prerequisites for ‘residence right’ through work, studies or with sufficient means to stay in the country for more than three months. Citizens of other countries need a residence permit issued by the Migration Agency [1]. As of the end of 2022, foreign-born persons who have immigrated and persons born in Sweden to two foreign-born parents constituted 20% and 26% of the Swedish population, respectively. Sweden reached a historical high in the number of new migrants in 2016 following the 2015 refugee ‘crisis’ [2]. The increasing numbers of migrants have given rise to frictions in Swedish society. The traditionally generous welcoming policies and attitudes toward migrants have become more hostile since the rise of populist parties [3, 4]. However, the Swedish Anti-Discrimination Act (2008:567) prohibits both direct and indirect discrimination as well as harassment based on gender, gender identity or expression, ethnic origin, religion or belief, disability, sexual orientation, and age [5].

Discrimination is defined by the United Nations Human Right Office of the High Commissioner as “any distinction, exclusion, restriction or preference based on race, colour, descent, ethnic origin, sex, age, gender, sexual orientation, gender identity, disability, religion or belief, nationality, migration or residence status or other status which has the purpose or effect of nullifying or impairing the recognition, enjoyment or exercise, on an equal footing, of human rights and fundamental freedoms in the political, economic, social, cultural or any other field of public life” [6]. Discrimination can be overt/direct or covert/indirect and may take three different forms: institutional, structural, and interpersonal discrimination [7]. Institutional discrimination occurs when state or non-state institutions applied unfair and discriminatory policies or practices. Structural discrimination refers to the ways in which society develops discrimination or policies that are neutral in intent but have negative effects on specific groups because of their arbitrary or ascribed characteristics that are socially attributed to belonging to a specific group based on traits as diverse as race, ethnicity, gender, religion or age. Interpersonal discrimination, on the other hand, refers to actions between individuals that are intended to have a differential impact whether individuals are in their institutional roles (e.g., care provider/patient) or as private individuals [7]. Regardless of its form, discrimination negatively affects people’s health and economic and social well-being [8–10]. Self-perceived discrimination can lead to negative consequences for both society and the victims such as school dropouts, social exclusion, ill health, avoidance or delay in seeking care and inadequate healthcare [10–12]. The same holds true for migrants, as discrimination can pose a significant challenge to their integration into receiving societies [12–15].

Discrimination against migrants can take place for different reasons and in different settings, including schools, labour markets, workplaces, public and health care services [12, 13, 16]. According to Schutze et al., preconceived notions about migrants can affect the welfare services that they receive in Sweden [17]. However, migrants consist of a diverse group of people from different countries and with different ethnic, religious, political, linguistic, and/or educational backgrounds, which may affect their vulnerabilities to discrimination and its consequences [12, 18, 19]. For instance, African-born and Muslim migrants are the groups most likely to be subjected to discrimination in European societies including Swedish society, which can pose a threat to their health, social integration, and well-being [3, 12]. However, most studies that have examined the harmful effects of discrimination on people’s health and well-being mainly focused on perceptions of racial or ethnic discrimination [8, 9, 20], only a few of these have focused on discrimination related to migrant or citizenship status [15, 18, 21] and far fewer have been conducted in Sweden [11, 22, 23].

Perceived discrimination from the host society is one of the main challenges to successful adaptation of migrants, as it reduces the possibility of adopting a positive attitude toward integration, and thus impedes successful adaption [12, 24, 25]. A Russian study found that perceived discrimination was strongly associated with poor psychological and sociocultural adaptation, which was partially mediated by the integration attitude among African migrants [15]. Another study found varying levels of adaptation among African migrants. Most migrants who reported a high level of perceived discrimination appeared to be relatively maladapted in Russian society, and a minority who perceived less discrimination regarded themselves as well-adapted and integrated [14]. Nevertheless, evidence from social science has revealed an ‘integration paradox’ suggesting that migrants and their descendants with apparently better access to mainstream middle-class society, as shown by their education, labour market success, length of residence, or generational status, often report more discrimination than those on the societal margins [26, 27].

Discrimination has negative effects on both mental and physical health and well-being, as documented by existing evidence [9, 18]. Self-reported racial/ethnic discrimination has been found to be associated with poorer self-rated health [8, 9, 28]. An earlier Swedish study found that high levels of anticipation of discrimination affected (self-rated) health [23]. Studies conducted in Canada also showed that discrimination or unfair treatment based on racial or ethnic discrimination (which also included language, accent, and religion) was an independent predictor of self-reported physical and mental health problems among new migrants and visible minorities [29, 30].

Migrants have limited access to health care services in receiving countries due to complex barriers, including discrimination [21, 31, 32]. Evidence shows that racial or ethnic discrimination can negatively affect access to care and treatment, trust in healthcare system [10, 33]. The few quantitative and qualitative studies conducted in Sweden have shown that perceived discrimination and socioeconomic disadvantage were independently associated with refraining from seeking medical treatment [33, 34]. Perceived institutional discrimination has been found to be associated with loss of trust in health care/health system and refraining from seeking care despite needs [34]. A study conducted in northern Sweden showed lower level of trust in healthcare among participants born outside of Sweden when compared to those born in the country. This disparity is likely connected to their adverse experiences with the healthcare system [35].

However, there is a scarcity of studies that have examined the prevalence and determinants of perceived discrimination. In addition, most earlier studies have almost exclusively focused on the association between discrimination and single outcomes. Studies that have simultaneously looked at the associations between perceived discrimination and different outcomes are scarce. As a result, the aim of this study was two-fold: [1] to investigate the prevalence, reasons and determinants of perceived discrimination among migrants attending Swedish language schools and [2] to explore the relationships between self-rated health, self-rated sexual health, use of health services, social integration, and perceived discrimination.

## Methods

### Study design, setting and participants

This study is exploratory in nature, driven by the limited availability of quantitative research in the field. We used data from a cross-sectional survey on migrants’ sexual and reproductive health and rights in Sweden conducted in 2018 (MSRHR-2018). The Public Health Agency of Sweden commissioned the MSRHR-2018 survey, following the national SRHR-2017 survey, as migrants are often underrepresented in national surveys [36].

The survey was conducted by the authors’ research team at Umeå university and took place at Swedish language and introductory programs schools. The survey was administered through visits to schools, by mail, and online. These schools were located in six of the 21 Swedish regions, representing different geographical areas: northern, central/middle, and southern. The target group was migrants residing in different parts of Sweden and aged 16 years or older. In this study the term migrant refers to all foreign-born individuals regardless of their country of birth, reason for migration, length of stay, and whether they have a residence permit or not. In this study, we focused on migrants from low- and middle-income countries and use.

### Survey instrument

The MSRHR-18 survey questionnaire was developed based on previous national and international surveys; the Sexuality and Health among Young People in Sweden (UngKab15) [37], Sexual and Reproductive Health and Rights in Sweden (SRHR17) [36] and the British National Survey of Sexual Attitudes and Lifestyles (Natsal-3) [38]. The MSRHR-18 survey has been described elsewhere [39]. The survey was available in Swedish and English and other four languages spoken by largest migrant communities (Arabic, Dari, Somali and Tigrinya) and consists of 71 questions relating to different issues including health, safety and social relationships, sexual and reproductive health and rights, and socio-demographic and socio-economic characteristics. Only questions relevant to the study aims were selected and included in this study.

### Data collection

The survey took place between 1 March and 30 September 2018 at schools, via mail and online. Initially, the school authorities were contacted via email or phone calls to request permission. Once permission was granted, the research team either visited the schools or sent the questionnaire with prepaid envelopes to teachers or other key persons. A convenience sampling strategy was used to recruit participants. All students present at schools on the days of the survey were asked to participate. The students answered the survey questionnaire anonymously in schools mainly by using a traditional paper-and-pencil method, or a computer in their preferred languages (their mother tongue, English, or Swedish). Bilingual project assistants from the students’ respective countries, teachers, or integration mentors assisted respondents with limited literacy or language skills. The online survey was administered through the Umeå University website and advertised through social media. The authors’ institution at Umeå University supervised and managed the data collection process.

**Fig. 1:**
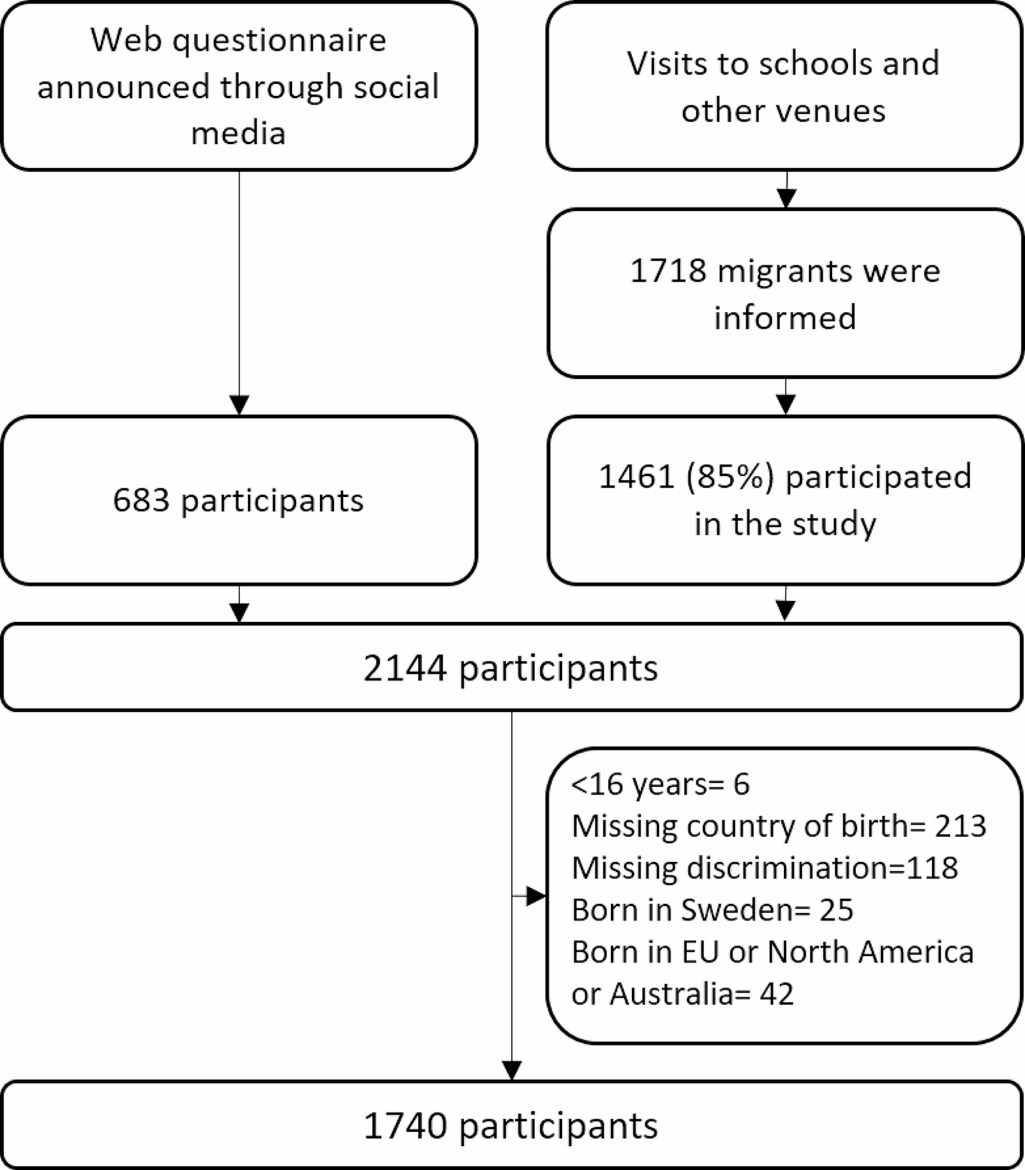
Flowchart of study participants

A total of 2144 migrants answered the survey questionnaire. These included 1461 out of the 1718 migrants who were informed/present at schools on the day of the survey administration and 683 who answered the questionnaire online. Unfortunately, we cannot provide the exact response rate due to the use of different recruitment and data collection methods, as well as the fact that the teachers did not provide us with the list of attendees. About one fifth (22%) answered the questionnaire in Swedish. The preferred languages for the remaining were Arabic (31%), Dari (18%), Tigrinya (12%), Somali (10%) or English (7%). Of the 2089 participants, 404 were excluded because they were born in Sweden (*n* = 25), other high-income countries (*n* = 42), did not answer the- question related to the main outcome variable perceived discrimination (118) or country of birth was missing (213). Finally, a total of 1740 was included in the analysis (See flowchart Fig. 1).

### Measures/variables

Relevant variables were selected from the MSRHR-18 survey to address the study aims 1 and 2.

#### Outcome variables

Perceived discrimination was used as the (primary) outcome variable to examine its association with socio-demographic characteristics (study aim 1). However, perceived discrimination was also used as an explanatory variable to address study aim 2. To assess perceived discrimination, respondents answered the following question: During the last 12 months in Sweden, have you been treated/addressed in a way that made you feel discriminated against or offended? The response options were dichotomized into *yes* (yes once or several times) and *no* (no).

For study aim 2, the following four variables were used as (secondary) outcomes: self-rated health, self-rated sexual health, refraining from seeking/visiting healthcare and/or social services regarding sexual or reproductive health issues, and self-perceived integration. To assess self-rated health and self-rated sexual health, respondents answered the following questions: How would you rate your general state of health and how would you rate your sexual health? The response options were dichotomized into *good* (very good or good) and *bad* (fair, poor or very poor). The following definition of sexual health from the World Health Organization was included in the information sheet and explained to respondents upon request: ‘Sexual health is a state of physical, mental, and social well-being in relation to sexuality. It requires a positive and respectful approach to sexuality and sexual relationships, as well as the possibility of having pleasurable and safe sexual experiences, free of coercion, discrimination, and violence’ [40]. Refraining from seeking care services was assessed by asking respondents the following question: In the previous 12 months, have you felt that you needed sexual or reproductive healthcare but did not seek care? The response options were ‘*yes’* and ‘*no’*. Self-perceived integration was assessed through the following question: How much do you feel as part of the Swedish society (sense of belonging) [41]? The response options were further dichotomized into *highly integrated* (fully integrated or to a greater extent) and *slightly or not integrated* (somewhat but not completely, slightly, not at all).

#### Independent variables

Independent variables included: region of birth, age, gender, sexual orientation, level of education, economic stress and language proficiency. Country of birth was grouped into four regions according to United Nations’ Regional groupings: Middle East and North Africa (MENA), Sub-Saharan Africa (SSA), South Asia (SA) and other regions. Age was grouped into four categories: 16–25, 26–35, 36–45 and 46 or older. Gender was categorized as man, woman and other. Sexual orientation was categorized as heterosexual, lesbian, gay, bisexual, asexual and other (LGBA+) and do not want to answer. Religion was categorized as Islam, Christianity, other (another religion, atheism, I don’t want to answer). Level of education was categorized as primary (≤ 9years), secondary (10–12 years) and tertiary (> 12 years). Economic stress was assessed by asking respondents the following question: During the last 12 months, have you ever had difficulty in managing the regular expenses for food, rent, bills etc.? The response options were further dichotomised as *yes* (yes once or more than once) and *no* (no). Language proficiency was assessed by asking respondents the following question: Do you need an interpreter when communicating with healthcare providers or other public services? The response options were dichotomised into *can communicate in Swedish* (No, I can speak Swedish) and *cannot communicate in Swedish* (yes always; yes sometimes; no, I can communicate in English; no, a family member helps me).

### Data analysis

We performed descriptive analysis to summarize the sample characteristics and estimate the prevalence of perceived discrimination, reasons and places of discrimination. Thereafter, we used log-binomial regression to compute crude and adjusted prevalence ratio of perceived discrimination to investigate its determinants. The regression was controlled for region of birth, age, gender, sexual orientation, religion, education level, economic stress, and language proficiency. To explore the relationships between self-rated health, self-rated sexual health, use of health services, self-perceived integration and perceived discrimination, we initially estimated the prevalence of these outcomes by perceived discrimination. Subsequently, we used used log-binomial regression to compute both crude and adjusted prevalence ratios of the associations between the four outcome variables and the exposure variable (perceived discrimination) using an outcome-wide epidemiological approach [42].We reported prevalence along with the 95% confidence intervals, considering significance at a P value below 0.05. The analysis was conducted using Stata 15 software. Microsoft Bing’s large language model was used to refine the English and condense the abstract.

## Results

### Sample characteristics and prevalence of perceived discrimination

The analytical sample consists of 1740 participants. Around 60% identified themselves as men. Slightly less than half were youth 16 to 25 years old (48%). The majority were heterosexual (68%), Muslim (61%) and could not communicate in Swedish (67%). Full sample characteristics are presented in Table 1.

Overall, about 36% of participants reported perceived discrimination. Migrants from the MENA (43%) and SA (49%) regions, those were youth (42%), identified themselves as men (41%) or LBGA+ (42%), had a tertiary level of education (39%), religious beliefs other (49%) than Christianity or Islam, experienced economic stress (50%) or could communicate in Swedish (44%) had the highest prevalence of perceived discrimination compared to their counterparts (See Table 1).

**Table 1:** Prevalence of perceived discrimination by sample characteristics

Characteristics	All (%)	Perceived Discrimination	
		No (%)	Yes (%)
**Total**	1,740 (100.0)	1,115 (64.1)	625 (35.9)
R**egion of birth**			
MENA	678 (39)	384 (56.6)	294 (43.4)
SA	360 (20.7)	184 (51.1)	176 (48.9)
SSA	592 (34)	453 (76.5)	139 (23.5)
Other	110 (6.3)	94 (85.5)	16 (14.6)
**Age**			
16 to 25	807 (48.4)	466 (57.7)	341 (42.3)
26 to 35	472 (28.1)	313 (66.3)	159 (33.7)
36 to 45	250 (14.9)	179 (71.6)	71 (28.4)
46 and older	151 (9)	123 (81.5)	28 (18.5)
**Gender**			
Woman	605 (37.4)	454 (75)	151 (25)
Man	969 (59.8)	573 (59.1)	396 (40.8)
Other	46 (2.8)	29 (63)	17 (37)
**Sexual orientation**			
heterosexual	1,017 (68.2)	649 (63.8)	368 (36.2)
LGBA	252 (16.9)	145 (57.5)	107 (42.5)
Don’t want to answer	222 (14.9)	155 (69.8)	67 (30.2)
**Religion**			
Islam	1,042 (61)	663 (63.6)	379 (36.4)
Christianity	397 (23.3)	294 (74.1)	103 (25.9)
Other	268 (15.7)	137 (51.1)	131 (48.9)
**Level of education (Years)**			
<=9	713 (42.9)	472 (66.2)	241 (33.8)
10 to 12	504 (30.3)	317 (62.9)	187 (37)
> 12	444 (26.7)	269 (60.6)	175 (39.4)
**Economic stress**			
No	964 (57.4)	715 (74.2)	249 (25.8)
Yes	717 (42.7)	361 (50.4)	356 (49.7)
**Language proficiency**			
Can communicate in Swedish	565 (32.9)	315 (55.8)	250 (44.3)
Cannot communicate in Swedish	1,151 (67.1)	785 (68.2)	366 (31.8)

### Reasons and places of discrimination

**Fig. 2:**
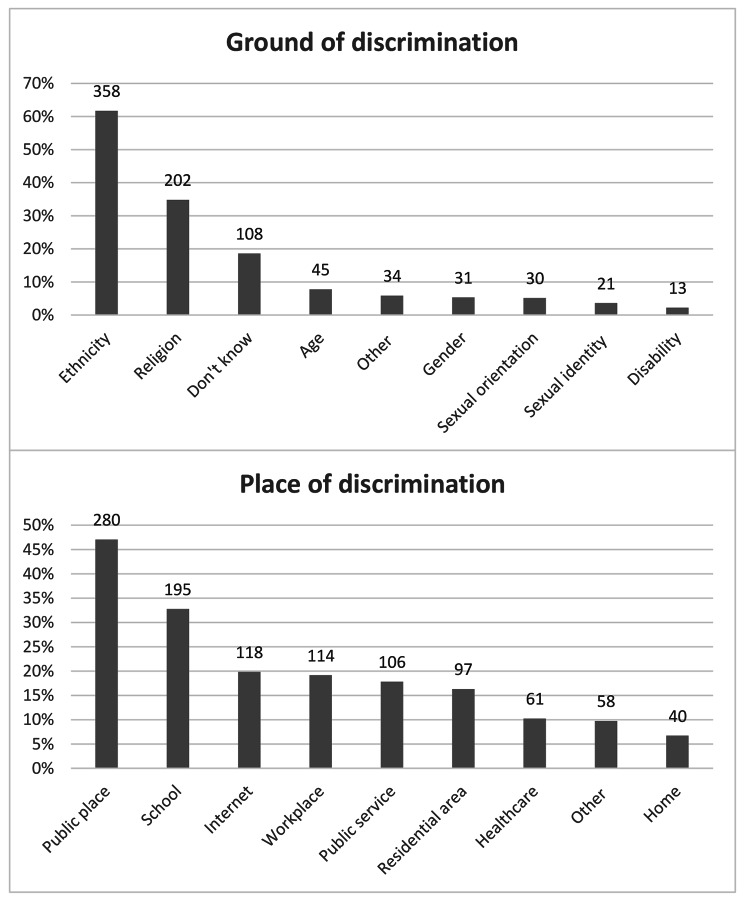
Reasons and places of discrimination

Ethnicity (62%) was the commonly reported reason of discrimination followed by religion (35%). Age (8%), gender (5%), sexual orientation (5%), disability (2%) and sexual identity (4%) were mentioned by less than 10% of participants. However, 19% of participants stated that they did not know why they were being discriminated against and another 6% responded that they were discriminated against for other reasons than the above-mentioned. The places where the discrimination occurred in descending order are public places (47%). schools (33%), internet (20%), workplace (19%), public services (18%), residential area (16%), healthcare settings (10%), other places (10%) and home (7%) (See Fig. 2).

### Association between perceived discrimination and socioeconomic and demographic characteristics

The bivariate analyses show associations between the prevalence ratio of self-perceived discrimination and region of birth, age, gender, religion, level of education, economic stress and language proficiency. All these associations as well as the association between perceived discrimination and sexual orientation were statistically significant in the multivariate analysis. The prevalence ratio of perceived discrimination was significantly higher among migrants from MENA and SA regions, migrant men, those who had a sexual orientation other than heterosexual, tertiary level of education, belonged to a religion other than Christianity or Islam, those who experienced economic stress, and those who could communicate in Swedish compared to the reference groups. The crude and adjusted prevalence ratios are presented in Table 2 below.

**Table 2:** Crude and adjusted prevalence ratios of the associations between perceived discrimination and socioeconomic and demographic characteristics

Socioeconomic and demographic characteristics	Crude PR (CI)*	Adjusted PR (CI)
**Region of birth**		
SSA	1	1
MENA	**1.85 (1.56 to 2.19)**	**1.57 (1.26 to 1.95)**
SA	**2.08 (1.74 to 2.49)**	**1.61 (1.27 to 2.04)**
Other	0.62 (0.38 to 1.00)	0.66 (0.39 to 1.09)
**Age (Years)**		
16 to 25	1	1
26 to 35	**0.80 (0.69 to 0.93)**	0.95 (0.80 to 1.13)
36 to 45	**0.67 (0.54 to 0.83)**	0.93 (0.75 to 1.17)
46 and older	**0.44 (0.31 to 0.62)**	**0.55 (0.37 to 0.80)**
**Gender**		
Woman	1	1
Man	**1.64 (1.40 to 1.92)**	**1.26 (1.07 to 1.49)**
Other	1.48 (0.99 to 2.21)	1.04 (0.67 to 1.60)
**Sexual orientation**		
Heterosexual	1	1
Other	1.17 (0.99 to 1.38)	**1.21 (1.02 to 1.43)**
Don’t want to answer	0.83 (0.67 to 1.04)	0.92 (0.73 to 1.15)
**Religion**		
Christianity	1	1
Islam	**1.40 (1.17 to 1.69)**	1.2 (0.96 to 1.51)
Other	**1.88 (1.53 to 2.32)**	**1.41 (1.10 to 1.80)**
**Level of education (Years)**		
<=9	1	1
10 to 12	1.10 (0.94 to 1.28)	1.17 (0.98 to 1.38)
> 12	**1.17 (1.00 to 1.36)**	**1.33 (1.10 to 1.60)**
**Economic stress**		
No	1	1
Yes	**1.92 (1.69 to 2.19)**	**1.67 (1.44 to 1.93)**
**Language proficiency**		
Cannot communicate in Swedish	1	1
Can communicate in Swedish	**1.39 (1.23 to 1.58)**	**1.24 (1.07 to 1.43)**

*PR: prevalence ratio, CI: confidence interval. In bold: Statistically significant (< 0.5)

### Prevalence of self-rated health, self-rated sexual health, refraining from seeking care and social integration by perceived discrimination

Participants who had experienced discrimination the last 12 months reported to a greater extent having bad general health (42.4% vs. 23.7 %) or sexual health (45.4% vs. 30.2%), being slightly or not at all integrated (70.3% vs. 52.4%) and refraining from seeking health services despite needs (27.5% vs. 14.2%) than those who did not (See Table 3).

**Table 3:** Prevalence of self-rated general and sexual health, perceived social integration and avoidance of healthcare services by perceived discrimination

Outcomes	All (%)	Perceived Discrimination	
		No (%)	Yes (%)
Total	1,740 (100.0)	1,115 (64.1)	625 (35.9)
**Self-rated general health**			
Bad	495 (30.2)	245 (23.7)	241 (42.4)
Good	1,145 (69.8)	817 (76.3)	328 (57.6)
**Self-rated sexual health**			
Bad	577 (35.7)	311 (30.2)	266 (45.4)
Good	1,040 (64.3)	720 (69.8)	320 (54.6)
**Self-perceived integration**			
Slightly or not all	1,001 (58.9)	570 (52.4)	431 (70.3)
Highly	699 (41.1)	517 (47.6)	182 (29.7)
**Refraining from seeking care despite needs**			
No	1,302 (80.9)	874 (85.8)	428 (72.5)
Yes	307 (19.1)	145 (14.2)	162 (27.5)

### The associations between self-rated general health, self-rated sexual health, refraining from seeking care and self-perceived integration and perceived discrimination

Both crude prevalence ratio (CPR) and adjusted prevalence ratio (APR) showed statistically significant associations between the four outcome variables and the exposure variable perceived discrimination. Participants who experienced discrimination had a significantly higher prevalence of bad self-rated general, bad self-rated sexual health, perceiving being slightly/not integrated, and refraining from seeking health services despite needs than those who did not experienced discrimination (See Table 4).

**Table 4:** Crude and adjusted prevalence ratios between self-rated general and sexual health, refraining from seeking care, self-perceived integration and perceived discrimination

Outcomes	Crude prevalence ratio	Adjusted* prevalence ratio
**Bad self-rated general health**		
Perceived discrimination No Yes	1 **1.79 (1.55 to 2.06)**	1 **1.72 (1.45 to 2.04)**
**Bad self-rated sexual health**		
Perceived discrimination No Yes	1 **1.50 (1.32 to 1.71)**	1 **1.40 (1.2 to 1.64)**
**Perceived being slightly or not integrated**		
Perceived discrimination No Yes	1 **1.34 (1.24 to 1.45)**	1 **1.25 (1.14 to 1.37)**
**Refrained from seeking** **health services despite needs**		
Perceived discrimination No Yes	1 **1.93 (1.58 to 2.36)**	1 **1.48 (1.16 to 1.89)**

*Adjusted for region of birth, age, gender, sexual orientation, religion, education, economic stress, and language proficiency. In bold: Statistically significant (P<:0.5)

## Discussion

This study shows that migrants in Sweden perceive discrimination in society, with ethnic origin and religion being the most frequently cited reasons for perceived discrimination. Perceived discrimination was reported to occur in various settings, including public spaces, schools, the internet, work, public services, residential areas, and healthcare settings. The study also reveals that migrant men, aged 16–25 years, born in MENA and SA regions, with more than 12 years of education, a sexual orientation other than heterosexual, religious beliefs other than Christianity, who reported economic stress or those who could communicate in Swedish perceived discrimination more frequently than their counterparts. Moreover, perceived discrimination was associated with poor self-rated general and sexual health, poor self-perceived integration, and avoidance of needed care.

### Pervasive and multiple forms of discrimination, but mainly ethnic and religious biases

Our study shows that there is a relatively high prevalence of perceived discrimination among migrants in Sweden, despite the Anti-Discrimination Act [5]. The prevalence found in this study is similar to what was reported in the second EU minorities and discrimination survey, but it is almost twice as high as that found in a recent study conducted in the United Kingdom involving the general population [12, 19]. A systematic analysis of media discourse on migration in Sweden from 2012 to 2019 has indicated that messages on social media generally had a negative tonality and suggest that some of the media frames can be attributed to a migration-hostile discourse [4]. While migrants can perceive discrimination on multiple grounds, region of birth (a proxy of ethnic origin) and religion are the most reported reasons [12]. In contrast, majority groups often mention gender and age as the common reasons for perceived discrimination [20].

### Some groups are more vulnerable than others

Our results reveal that the prevalence of perceived discrimination varies by socioeconomic and demographic characteristics, with some groups perceiving more discrimination than others, reflecting the diversity of the migrant population [12, 18, 19]. For instance, migrants with a sexual orientation other than heterosexual are very likely to experience double discrimination and marginalization by compatriots, authorities, immigration officials, polices due to their migrant or minority status and their sexual orientation [43]. However, the high proportion of participants who did not identify themselves as heterosexuals warrants additional investigation as they barely represented 3% in the national MSRHR-2017 survey [36]. Sweden may be attractive to LGBTQI + migrants fleeing persecution in their home countries as they can be granted protection according to Swedish law. A qualitative study involving LGBTQI + migrants living with HIV in Sweden suggested that current legislation in Sweden makes it a safe and secure country where LGBTQI people can live a normal life and access HIV treatment without discrimination [13]. Previous research has also shown that self-perceived discrimination is related to gender, ethnic origin and religion [12, 18]. For instance, Muslim men perceive being subject to gendered stereotypes such as being unfairly suspected of being abusive, patriarchal fathers and potential perpetrators of honor violence [16]. Men with Arabic names are also reported to face greater discrimination than women with Arabic names in the labor market in Sweden [44] and Denmark [45]. Bursell (2021) has argued that women may more easily escape negative characterizations or stereotypes than men if certain nationalities or ethnic groups are disliked for some reason [16]. On the other hand, migrants in the youngest age group may have greater exposure to the new community (e.g. schools, social activities), and therefore more likely to perceive or experience discrimination more frequently compared to those in the oldest age group [12]. For example, Muslim girls in Sweden complain of being stereotyped as both victims and threats. They are portrayed as victims who need to be saved from their patriarchal families that imposed Islam upon them. Meanwhile, they are also stereotyped as ‘cultural’ threats, embodying Islam. Muslim boys, on the other hand, complain of being perceived as under-performing, threatening troublemakers in the Swedish educational system [16].

Furthermore, despite the lack of statistically significant difference in the prevalence of perceived discrimination between Christians and Muslims after adjustment in the multivariate analysis, our results indicate that there is a societal reluctance to tolerate religions other than Christianity. This creates a paradox for non-Christian migrants. Sweden is considered one of the most secular societies in the world, yet it has a strong Lutheran heritage [46]. Additionally, perceived discrimination was associated with regions of birth (MENA and SA) where Islam is the dominant religion. This may indicate an association/interaction between religion and region of birth that has influenced the association between religion and perceived discrimination in the multivariate analysis. Moreover, the category ‘other’ for religion includes atheists, those with another religion, and those who were unwilling to answer. In Sweden, Islam and Muslims have been portrayed as a threat to the country’s cherished values of secularism, gender equality, democracy, and the welfare state. The issue of terrorism has emerged as a key concern in Swedish public opinion on Muslims, particularly in the wake of terror attacks in other Western countries and on Swedish soil, leading to widespread generalizations and stereotypes about Islam and Muslims, amounting to “Islamophobia” [47]. For example, a study found that 30% of respondents in the Diversity Index (2016) stated that they would move to another neighborhood if ‘too many’ people from Muslim countries were to move in, while 18% felt the same way about neighbors of African origin, and only 5% felt that way about Europeans. Swedish Muslims have also confirmed that they perceive these negative attitudes in other studies [16]. Likewise, nearly one in five (16%) participants in this study perceived discrimination in residential areas.

Our findings further support the integration paradox. According to Schaeffer et al. (2023) while better education and language proficiency suggest easier integration into mainstream society, they may also lead to greater exposure to mainstream members, heightened familiarity with exclusionary public discourse, or a heightened risk of downward social mobility or competition with natives on the labor market. These mechanisms are expected to increase cognitive susceptibility to framing experiences in terms of discrimination or simply the opportunities to encounter discrimination, which in turn is expected to increase reports of discrimination [26]. On the other hand, the positive association between perceived discrimination and economic stress could be partly explained by the fact that Non-western migrants are overrepresented among socioeconomically disadvantaged ethno-racial groups in Sweden [48]. As a result, they are often stereotyped as a burden and a threat to the welfare system and are likely to be over-scrutinized and unfairly suspected of free-riding behavior (i.e. unworthy claimants of the resources and services of the welfare state) [47], which results in them receiving fewer resources and opportunities than other groups [16]. This perpetuates inequality and places an undue burden on them.

### Perceived discrimination is not limited to public spaces, also common within public institutions

The perceived discrimination reported in this study is not limited to public spaces, but also occurs within public institutions such as public services, schools, and healthcare settings. Likewise, a previous study on complaints to the Swedish Ombudsman against Discrimination found that 50% targeted the educational system, 15% the judicial system, and 10% each for healthcare institutions, social security, and social insurance related agencies. The remaining 5% were directed at other institutions. The complaints concerned perceptions of institutional rules as ethnocentric or discriminatorily inflexible and being distrusted and over-scrutinized, more harshly judged i.e., being denied, or cut off from resources or opportunities, neglected, obstructed, and harassed [16]. Perceived discrimination within public institutions can result from rules that are neutral to ethnicity, but that public officials also known as street-level bureaucrats, apply in a biased manner due to ethnocentric and essentialist views of minority culture [49]. Evidence suggests that ethno-racial minorities are more likely to receive welfare sanctions in the US and to be denied social insurance benefits, stopped or subject to discriminatory profiling by the police in Sweden [12, 50]. Experiences of discrimination within healthcare have also been reported in various settings [21, 31, 32]. Such discrimination is worrisome as it can contribute to a distrust in public institutions and reluctance to seek available services and support, and result in unequal opportunities in accessing these services between migrant and non-migrant groups [12].

### Perceived discrimination related to Health, Healthcare and Integration challenges

Not surprisingly, self-perceived discrimination was associated with health, healthcare and integration challenges. The association between perceived racial/ethnic discrimination and poor health outcomes among migrants has been largely reported in other studies [18, 23, 28]. However, the association between poor self-rated sexual health and perceived discrimination found in this study needs further investigation, as it might be mediated by other factors such as sexual orientation. The association between experiences of ethno-racial discrimination and access to healthcare has also been highlighted in several studies. Perceived ethno-racial discrimination has been identified as a barrier to care for migrants that reinforce inequities in healthcare access and quality for racialized migrants in several studies in Sweden and elsewhere [21, 31, 32, 34].

Perceived discrimination from the receiving culture has also been recognized as one of the main acculturation challenges that hinders successful adaptation of migrants in general [12, 24, 25]. In Sweden, political and public debates have primarily focused on the societal and economic challenges associated with increased migration, with less attention given to the challenges faced by individuals with migrant backgrounds themselves. While Sweden has been ranked as the country with the most integration-promoting policies in the world, research has shown that such positive policies may not align with the everyday experiences of migrants [51, 52]. Qualitative studies suggest that experiences of prejudice and discrimination are the most commonly occurring themes among migrants when discussing their experiences of life in Sweden [13, 51]. Most respondents in a Russian study of African migrants’ sociocultural adaptation reported experiencing a high level of perceived discrimination and appeared to be relatively maladapted in Russian society. However, those who regarded themselves as “well-adapted” were more likely to perceive native Russians’ attitudes toward them as positive or tolerant, indicating that they experienced less discrimination [14]. According to Ivande & Ryabichenko (2023), perceived discrimination not only directly hinders adaptation but also indirectly affects acculturation attitudes by reducing the likelihood of adopting a positive attitude toward integration, thereby impeding successful adaptation [15].

### Strengths and limitations

This study has several strengths. It included a relatively large sample size of participants fluent in at least one of the six languages used in the survey and spoken by the largest migrant communities in Sweden. Participants with limited literacy had the possibility of personal assistance during face-to-face data collection by people speaking their mother tongue. The survey was administered both in schools and other venues and online to reach different groups of migrants, which enabled us to include migrants with different backgrounds and expand the diversity of participants.

However, there are some potential challenges in our sample. The participants were selected conveniently, and some participants could have been excluded due to language barriers. Even though we believe that the characteristics of the sample regarding gender and region of birth reflect the structure of newly arrived migrants to Sweden, it is difficult to generalize to other groups of migrants as the findings may only reflect the situation among newly arrived migrants. The study used a cross-sectional design and asked about discrimination in the past year, making recall bias a potential problem. Moreover, privacy concerns could have influenced responses collected in schools or homes, despite measures to ensure privacy during data collection. We also acknowledge the potential for social desirability/respondents’ bias in self-report questionnaires. To mitigate this, we employed anonymous self-administration and emphasized to participants that there were no wrong or right answers, aiming to enhance the likelihood of obtaining more truthful responses. Additionally, we utilized simple wording and forced-choice items that compelled respondents to provide specific answers for questions related to the main outcome variables. While acknowledging the uncertain validity of the self-rated sexual health item, it is crucial to recognize the WHO’s definition of sexual health as a multifaceted concept beyond measurable physical and mental abnormalities. Therefore, multiple indicators are needed to assess different aspects of sexual health. Individuals, when rating their health, consider various aspects beyond physical symptoms. In this study, we were interested in individual experiences of sexual health, exploring how individuals interpret and synthesize these different facets. Another limitation of the cross-sectional design and exploratory nature of this study is the issue of temporality making causality difficult to assume. This underscores the need for an explanatory approach to assess pathways and intersectionality.

Despite these limitations, the study provides valuable insights in understanding the extent of perceived discrimination, reasons why and places where it happens and potential consequences among migrants in Sweden.

## Conclusions

This study highlights the pervasive issue of perceived discrimination against migrants in Swedish society, which affects them in various settings and contexts. Ethnic origin and religion emerge as primary reasons for perceived discrimination, but certain groups are more vulnerable than others. The findings also indicate associations between poor health outcomes, poor self-perceived integration, avoidance of needed care, and perceived discrimination, suggesting a detrimental effect on well-being, participation, sense of belonging, integration efforts, and health and healthcare disparities. These findings emphasize the urgent need for policies and interventions that address systemic discrimination (i.e., ethnic and religious biases prevalent in the society), promote inclusive environments (promote integration), and ensure equitable access to healthcare and resources for migrants in Sweden. Targeted programs and an intersectional approach to support vulnerable groups perceiving heightened discrimination, such as migrants from certain regions or those experiencing economic stress, are also crucial. Further research is warranted to investigate intersectional discrimination and mediation pathways between discrimination and various health outcomes, as well as other related outcomes.

## Data Availability

No datasets were generated or analysed during the current study.

## Abbreviations

APR: Adjusted prevalence ratio
CPR: Crude prevalence ratio
EEA: European Economic Area
EU: European Union
LGBA +: Lesbian, gay, bisexual, asexual and other
MENA: Middle East and North Africa
MSRHR-2018: Migrants’ sexual and reproductive health and rights 2018 survey
SA: South Asia
SAA: Sub-Saharan Africa

## References

[1] Statistics Sweden. Invandring till Sverige-Vem räknas som invandrare? Statistikmyndigheten.; 2023. Available from: https://www.scb.se/hitta-statistik/sverige-i-siffror/manniskorna-i-sverige/invandring-till-sverige/.

[2] Statistics Sweden. Utrikes födda i Sverige: Statistikmyndigheten.; 2023. Available from: https://www.scb.se/hitta-statistik/sverige-i-siffror/manniskorna-i-sverige/utrikes-fodda-i-sverige/#utrikes-fodda-over-tid.

[3] Puschmann P, Sundin E, De Coninck D, d’Haenens L. Migration and integration policy in Europe: Comparing Belgium and Sweden. Images of immigrants and refugees in Western Europe: Media representations, public opinion and refugees’ experiences. 2019:21–36.

[4] Yantseva V (2020). Migration discourse in Sweden: frames and sentiments in mainstream and social media. Social Media + Soc [Internet].

[5] Regeringskansliets rättsdatabaser. Diskrimineringslag (2008:567): Svensk författningssamling (SFS).; 2022. Available from: https://lagen.nu/2008:567.

[6] United Nations Human Rights Office of the High Commissionner (OHCHR). Principles and Guidelines, supported by practical guidance, on the human rights protection of migrants in vulnerable situations: UN Office of the High Commissioner for Human Rights (OHCHR) & Global Migration Group (GMG).; 2017. Available from: https://www.ohchr.org/sites/default/files/Documents/Issues/Migration/PrinciplesAndGuidelines.pdf.

[7] Pincus FL (1996). Discrimination comes in many forms: individual, institutional, and structural. Am Behav Sci.

[8] Brondolo E, Hausmann LR, Jhalani J, Pencille M, Atencio-Bacayon J, Kumar A (2011). Dimensions of perceived racism and self-reported health: examination of racial/ethnic differences and potential mediators. Ann Behav Med.

[9] Paradies Y, Ben J, Denson N, Elias A, Priest N, Pieterse A (2015). Racism as a determinant of health: a systematic review and meta-analysis. PLoS ONE.

[10] Chen D, Yang T-C (2014). The pathways from perceived discrimination to self-rated health: an investigation of the roles of distrust, social capital, and health behaviors. Soc Sci Med.

[11] Bursell M, Bygren M, Gähler M (2021). Does employer discrimination contribute to the subordinate labor market inclusion of individuals of a foreign background?. Soc Sci Res.

[12] European Union Agency for Fundamental Rights (FRA). Second European Union Minorities and Discrimination Survey: FRA.; 2017. Available from: https://fra.europa.eu/sites/default/files/fra_uploads/fra-2017-eu-midis-ii-main-results_en.pdf.

[13] Nkulu-Kalengayi FK, Ouma AA, Hurtig A-K (2022). HIV ended up in second place’– prioritizing social integration in the shadow of social exclusion: an interview study with migrants living with HIV in Sweden. Int J Equity Health.

[14] Bondarenko DM. African Migrants in Post-Soviet Moscow: Adaptation and Integration in a Time of Radical Socio-Political Transformations. Ìrìnkèrindò: A Journal of African Migration, United States. 2017;9:34–71.

[15] Ivande SK, Ryabichenko T (2023). The relationship between perceived discrimination, acculturation attitudes, and adaptation among anglophone African immigrants in Russia: the moderating role of neuroticism. Psychol Russia: State Art.

[16] Bursell M (2021). Perceptions of discrimination against muslims. A study of formal complaints against public institutions in Sweden. J Ethnic Migration Stud.

[17] Schütze C (2020). Attitudes matter—welfare work and migration in Sweden. Migration Stud.

[18] Schunck R, Reiss K, Razum O (2015). Pathways between perceived discrimination and health among immigrants: evidence from a large national panel survey in Germany. Ethn Health.

[19] Maletta RM, Daly M, Goodwin L, Noonan R, Putra IGNE, Robinson E (2023). Prevalence of perceived discrimination and associations with mental health inequalities in the UK during 2019–2020: a cross-sectional study. Psychiatry Res.

[20] Gyberg F, Svensson Y, Wängqvist M, Syed M (2021). Discrimination and its relation to psychosocial well-being among diverse youth in Sweden. New Dir Child Adolesc Dev.

[21] Rivenbark JG, Ichou M (2020). Discrimination in healthcare as a barrier to care: experiences of socially disadvantaged populations in France from a nationally representative survey. BMC Public Health.

[22] Groglopo A, Ahmadi F, Munobwa JS (2023). Structural racism in Sweden: framing attitudes towards immigrants through the Diversity Barometer Study (2005–2022). Social Sci.

[23] Mohseni M, Lindström M (2008). Ethnic differences in anticipated discrimination, generalised trust in other people and self-rated health: a population-based study in Sweden. Ethn Health.

[24] Güler A, Yıldırım M (2022). Associations between acculturation, perceived discrimination and subjective well-being among Syrian adolescents living in Turkey. Int J Psychol.

[25] Hashemi N, Marzban M, Sebar B, Harris N (2019). Acculturation and psychological well-being among middle eastern migrants in Australia: the mediating role of social support and perceived discrimination. Int J Intercultural Relations.

[26] Schaeffer M, Kas J. The integration paradox: a review and meta-analysis of the complex relationship between integration and reports of discrimination. Int Migrat Rev. 2023:01979183231170809.

[27] Verkuyten M (2016). The integration paradox: empiric evidence from the Netherlands. Am Behav Sci.

[28] Tsuchiya K, Schulz AJ, Niño MD, Caldwell CH. Perceived Racial/Ethnic discrimination, citizenship status, and self-rated health among immigrant young adults. J Racial Ethnic Health Disparities. 2023:1–13. 10.1007/s40615-023-01731-1 PMC11134944 37566180

[29] De Maio FG, Kemp E (2010). The deterioration of health status among immigrants to Canada. Glob Public Health.

[30] Fuller-Thomson E, Noack AM, George U (2011). Health decline among recent immigrants to Canada: findings from a nationally-representative longitudinal survey. Can J Public Health.

[31] Hamed S, Bradby H, Ahlberg BM, Thapar-Björkert S (2022). Racism in healthcare: a scoping review. BMC Public Health.

[32] Pattillo M, Stieglitz S, Angoumis K, Gottlieb N (2023). Racism against racialized migrants in healthcare in Europe: a scoping review. Int J Equity Health.

[33] Hamed S, Thapar-Björkert S, Bradby H, Ahlberg BM (2020). Racism in European health care: structural violence and beyond. Qual Health Res.

[34] Wamala S, Merlo J, Boström G, Hogstedt C (2007). Perceived discrimination, socioeconomic disadvantage and refraining from seeking medical treatment in Sweden. J Epidemiol Community Health.

[35] Baroudi M, Goicolea I, Hurtig A-K, San-Sebastian M (2022). Social factors associated with trust in the health system in northern Sweden: a cross-sectional study. BMC Public Health.

[36] The Public Health Agency of Sweden. Sexuell och reproduktiv hälsa och rättigheter i Sverige 2017. Stockholm: Folkhälsomyndigheten.; 2019. Available from: http://www.folkhalsomyndigheten.se.

[37] The Public Health Agency of Sweden (2017). Sexualitet och hälsa bland unga I Sverige–UngKAB15–en studie om kunskap, attityder och beteende bland unga 16–29 år.

[38] Erens B, Phelps A, Clifton S, Mercer CH, Tanton C, Hussey D et al. Methodology of the third British national survey of sexual attitudes and lifestyles (Natsal-3). Sexually transmitted infections. 2013. 10.1136/sextrans-2013-051359 PMC3933071 24277881

[39] Baroudi M, Kalengayi FN, Goicolea I, Jonzon R, San Sebastian M, Hurtig A-K (2022). Access of migrant youths in Sweden to sexual and reproductive healthcare: a cross-sectional survey. Int J Health Policy Manage.

[40] World Health Organization. Defining Sexual Health: Report of a technical consultation on sexual health, 28–31 January 2002, Geneva 2006. Available from: http://www.who.int/reproductivehealth/publications/sexual_health/defining_sexual_health.pdf.

[41] Wu Z, Schimmele CM, Hou F (2012). Self-perceived integration of immigrants and their children. Can J Sociol.

[42] VanderWeele TJ (2017). Outcome-wide epidemiology. Epidemiol (Cambridge Mass).

[43] Yarwood V, Checchi F, Lau K, Zimmerman C (2022). LGBTQI + migrants: a systematic review and conceptual framework of health, safety and wellbeing during migration. Int J Environ Res Public Health.

[44] Arai M, Bursell M, Nekby L (2016). The reverse gender gap in ethnic discrimination: employer stereotypes of men and women with arabic names. Int Migrat Rev.

[45] Dahl M, Krog N (2018). Experimental evidence of discrimination in the labour market: intersections between ethnicity, gender, and socio-economic status. Eur Sociol Rev.

[46] Kasselstrand I (2015). Nonbelievers in the church: a study of cultural religion in Sweden. Sociol Relig.

[47] Mulinari D, Neergaard A (2012). Violence, racism, and the political arena: a scandinavian dilemma. NORA-Nordic J Feminist Gend Res.

[48] Gustafsson B, Zheng J (2006). Earnings of immigrants in Sweden, 1978 to 1999. Int Migration.

[49] Eliassi B (2017). Conceptions of immigrant integration and racism among social workers in Sweden. J Progressive Hum Serv.

[50] Social Insurance Inspectorate. (*Inspektionen för socialförsäkringen (ISF*). Foreign Born Individuals are More Often Denied Sickness Benefits than Native Born Individuals (*Nekad sjukpenning för inrikes och utrikes födda*). Stockholm2016. Available from: https://isf.se/download/18.6e75aae16a591304896bcf/1565330425495/Nekad%20sjukpenning%20fo%CC%88r%20inrikes%20och%20utrikes%20fo%CC%88dda-ISF-Rapport%202016-05.pdf.

[51] Gyberg F, Frisén A, Syed M, Wängqvist M, Svensson Y (2018). Another kind of Swede Swedish Youth’s ethnic identity narratives. Emerg Adulthood.

[52] Migration and Integration Policy Index (MIPEX). Sweden: MIPEX.; 2020. Available from: https://www.mipex.eu/sweden.

